# A case report and brief review of the literature on bilateral retinal infarction following cardiopulmonary bypass for coronary artery bypass grafting

**DOI:** 10.1186/1749-8090-6-154

**Published:** 2011-11-21

**Authors:** Brian A Trethowan, Helen Gilliland, Aron F Popov, Barathi Varadarajan, Sally-Anne Phillips, Louise McWhirter, Robert Ghent

**Affiliations:** 1 Department of Anaesthesia, Royal Group of Hospitals and Dental Hospital Health and Social Services Trust, Grosvenor Road, Belfast, BT12 6BA, Northern Ireland; 2 Department of Cardiothoracic Transplantation & Mechanical Support, Royal Brompton & Harefield NHS Trust, Harefield, UB9 6JH, London, United Kingdom; 3 Department of Critical Care, The Royal London Hospital, Whitechapel Road, E1 1BB, London, United Kingdom

## Abstract

Postoperative visual loss is a devastating perioperative complication. The commonest aetiologies are anterior ischaemic optic neuropathy (AION), posterior ischaemic optic neuropathy (PION), and central retinal artery occlusion (CRAO). These appear to be related to certain types of operation, most commonly spinal and cardiac bypass procedures; with the rest divided between: major trauma causing excessive blood loss; head/neck and nasal or sinus surgery; major vascular procedures (aortic aneurysm repair, aorto-bifemoral bypass); general surgery; urology; gynaecology; liposuction; liver transplantation and duration of surgery. The non-surgical risk factors are multifactorial: advanced age, prolonged postoperative anaemia, positioning (supine v prone), alteration of venous drainage of the retina, hypertension, smoking, atherosclerosis, hyperlipidaemia, diabetes, hypercoagulability, hypotension, blood loss and large volume resuscitation. Other important cardiac causes are septic emboli from bacterial endocarditis and emboli caused by atrial myxomata. The majority of AION cases occur during CPB followed by head/neck surgery and prone spine surgery. CPB is used to allow coronary artery bypass grafting on a motionless heart. It has many side-effects and complications associated with its use and we report here a case of bilateral retinal infarction during routine coronary artery bypass grafting in a young male patient with multiple risk factors for developing this complication despite steps to minimise its occurrence.

## Case Report

A 36 year-old Caucasian male (59 kg, BMI 23.8 kg.m^-2^) presented for urgent coronary artery bypass grafting 4 weeks after admission to the coronary care unit with a non-ST elevation myocardial infarction. He had episodic chest pain for 1 year increasing in frequency over six weeks prior to admission. On admission to hospital he was commenced on medication including acetylsalicylic acid, Clopidogrel, Elantan LA, Bisoprolol, Ramipril, Ezetimibe and Enoxaparin and he remained as an inpatient until his scheduled surgical procedure without requiring heparin, GTN infusion or inotropes. Urgent coronary angiography revealed a critical lesion of the left main stem artery, 70% stenosis of the proximal circumflex and 70% stenosis of the origin of the posterior descending artery and moderate impairment of left ventricular function with inferoposterior hypokinesis. This impairment in left ventricular was confirmed by transthoracic echocardiography. On arrival in the anaesthetic room his initial blood pressure was recorded as 90/50 mmHg and this had no apparent effect on pre-operative organ function considering all blood tests were within normal range and urine output was >1 ml.kg^-1^.hr^-1^. General anaesthesia was induced and was further maintained with propofol target-controlled infusion (1.5μg.ml^-1^), remifentanil (0.34μg.kg^-1^.min^-1^) and isoflurane/oxygen/air mix at FiO_2 _of 0.4 and end-tidal isoflurane 0.4-0.6%. Peri-operative transoesophageal echocardiography was used as part of monitoring in this case and epi-aortic ultrasound scanning was not utilised. As the left internal mammary artery was being taken down a dose of 250 mg (4.3 mg/kg) of heparin was given with a subsequent ACT of 559s. During CPB mean arterial pressure was maintained between 50-60 mmHg using boluses of phenylephrine and subsequently an infusion of noradrenaline. The lowest recorded mean arterial pressure was 45 mmHg immediately post institution of CPB. The infusion of noradrenaline ran at doses from 0.05-0.075μg.kg^-1^.min^-1 ^throughout the procedure. Other drugs that were used include glyceryl trinitrate at 0.5 mg.hr^-1 ^as well as an infusion of the synthetic anti-fibrinolytic tranexamic acid initially at 1000 mg.hr^-1 ^reduced to 600 mg.hr^-1 ^(0.2 mg.kg^-1^.min^-1^) to reduce perioperative blood loss. He received an infusion of milrinone at a dose 2μg.kg^-1^.min^-1^. Mixed venous saturation ranged from 55-70% in the peri-operative period. Pre-operatively the Hb concentration was 11.5 g.dl^-1^. Intra-operatively the range of Hb was 7.5-10.5 g.dl^-1 ^with the nadir on admission to CSICU. He was cooled and reached a nadir of 30°C. He was successfully weaned from CPB after placement of an intra-aortic balloon pump (IABP) and continuation of vasopressor and inotropic support. On admission to the Cardiac Surgical intensive care unit (CSICU) his mean blood pressure was 60 mmHg with a mean PA pressure of 14 mmHg. The balloon pump counter-pulsation remained on a 1:1 ratio with noradrenaline 0.05μg.kg^-1^.min^-1^, dopamine 5μg.kg^-1^.min^-1^, adrenaline 0.075μg.kg^-1^.min^-1^, and milrinone 1.5μg.kg^-1^.min^-1 ^to maintain a mean pressure of 75 mmHg, CI> 2.2 and adequate urine output >0.5 ml.kg^-1^.hr^-1^. The Hb concentration immediately on admission to CSICU post-operatively was 6.4 g.dl^-1^, he was transfused with 3 units of packed cells and thereafter it remained between 8-10 g.dl^-1^. On arrival in CSICU it was noted that his pupils were fixed and dilated at 5 mm. His intraocular pressure (IOP) was measured and found to be 13 mmHg. The measure of intraocular pressure served to rule out glaucoma and was a surrogate for measurement of intracranial pressure (ICP). During a sedation hold he was able to respond to command appropriately. The intra-aortic balloon pump was weaned quickly post-operatively and removed just over 12 hours after admission to CSICU. On the second day post-operatively whilst being turned he suffered a brief asystolic arrest requiring approximately 1 minute of CPR before return of spontaneous circulation, with similar pre-arrest haemodynamics. There was no warning of this from haemodynamic monitoring and on review of the charts the acute nature of the incident would appear to fit with a failure of pacing capture. However, subsequent urgent transesophageal echocardiography showed non-compressive clot and fluid around the right atrium, with CVP of 10-12 and mean PAP 16 mmHg. He returned to theatre for a re-exploration of his pericardium at which time a small amount of clot was removed and no revision of pacing wires was required. Over the next 36 hours he was weaned from all inotropic and vasopressor medications. He was extubated on day 3 post-operatively, had normal vision at this stage and was able to see staff within CSICU despite still having fixed and dilated pupils. He was re-intubated on day 5 after a failed trial of non-invasive therapy for bibasal collapse and worsening oxygenation. 7 days post-operatively, after being extubated for the second time, the patient complained of poor vision. An urgent ophthalmology review was organised and their findings were as follows: visual acuity showed no perception of light bilaterally, both pupils fixed and dilated, unreactive to light or accommodation, no consensual response, normal intraocular pressure, good red reflex, bilateral disc pallor, indistinct disc margins, retinal pallor at posterior pole, cattle-trucking of retinal arterial circulation. He also had bilateral internuclear ophthalmoplegia with up-gaze and down-gaze weakness. The impression was one of bilateral anterior ischaemic optic neuropathy (AION) and impending bilateral central retinal artery occlusion (CRAO) with brain stem involvement. A CT brain showed marked calcification of the falx, tentorium and choroid plexus with a further abnormality in the corpus callosum representing an unusual area of focal ischaemia (most likely in keeping with recent cardiac surgery). Rescue therapy with acetazolamide and topical timolol to reduce IOP and improve intraocular perfusion pressure, despite normal IOP, was attempted, but there was no improvement in vision. During follow up by neurology and ophthalmology services within the hospital he underwent a MRI of his brain with Magnetic Resonance Angiography of his cerebral vessels. No white matter abnormality was identified within cerebral hemispheres and the brainstem, cerebellum and pituitary fossa were unremarkable. He was also referred to a clinical psychologist and the Royal National Institute for the Blind (RNIB) for support and rehabilitation.

## Discussion

The incidence of postoperative visual loss ranges from 0.0008%-0.002% in non-ocular, non-cardiac surgery to 0.06-4.5% in CPB series [1-9]. The most common post-operative visual loss defects are related to ischaemic optic neuropathy (ION) CRAO and cortical blindness [1]. There is still much controversy surrounding this injury and it appears it is not always preventable. Should we however inform all patients of the risk given the incidence of post-operative visual loss is rare but devastating? The lack of certainty of the mechanism of the insult limits our understanding combined with the multiplicity of risk factors. The rodent model described by Bernstein is of uncertain importance as most evidence is from case-series or case-control studies in cardiac, spinal, trauma and non-ocular surgery [2,4-6,8,10,11].

**Figure 1:**
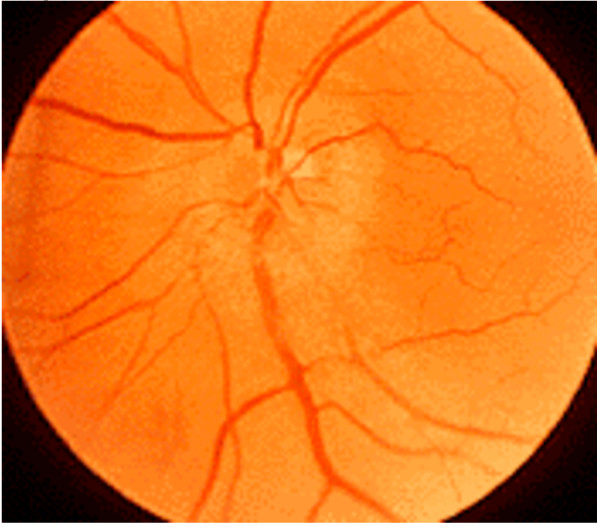
**Early AION (note oedematous fundal appearance)**.

**Figure 2:**
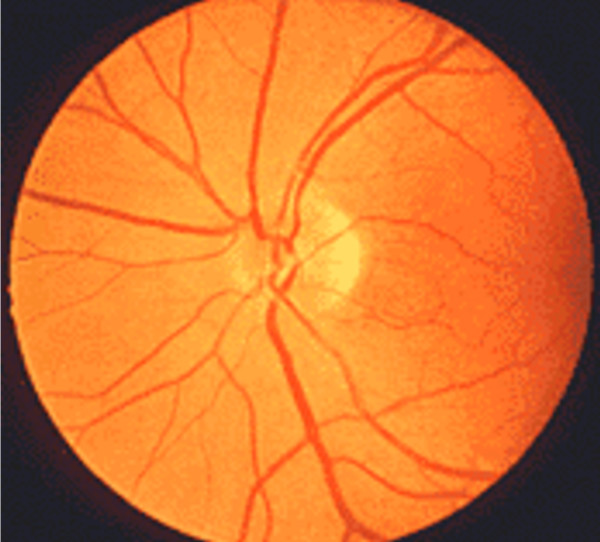
**Normal fundus and early PION (note normal fundal appearance)**.

**Figure 3:**
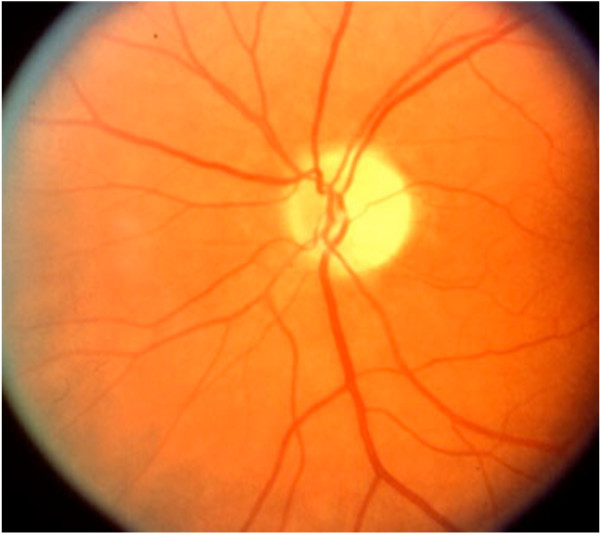
**Late AION and PION (note pallor of fundal appearance)**.

In this case report, we will restrict our discussion to the aetiology and mechanisms pertaining to ION describing the clinical findings and mechanism of both AION and PION. AION occurs at the optic nerve head as the optic nerve and retinal vessels enter the globe. PION occurs anywhere posterior from the optic nerve head to the optic chiasm. Early fundoscopic examination will demonstrate disc oedema in AION (Figure 1), whereas the fundus is entirely normal in early PION (Figure 2). Days to weeks later fundoscopy reveals optic nerve pallor in both conditions (Figure 3). Pupillary light reflexes are absent or delayed in both conditions and patients may complain of total blindness or altitudinal field defects (visual loss above or below the visual horizon). Delayed or worsening visual loss usually implicates AION as the probable diagnosis and the delay can be up to a few weeks after surgery.

In just over half of the ION cases in the American Society of Anaesthesiologists Post Operative Visual Loss (ASA POVL) Registry, both eyes were affected, suggesting a systemic event [1]. Complete recovery from either AION or PION is rare.

AION has been primarily associated with CPB procedures but can also occur during prone spine operations and spontaneously. PION is most commonly associated with lengthy prone spine operations and bilateral radical neck operations. Numerous contributory factors for AION and PION have been proposed (hypotension, anaemia, oedema, venous congestion, variations in ocular anatomy and physiology, effects of fluid resuscitation) but none has been causally linked [11,12]. Older patients are more at risk of ION due to the natural reduction in nerve fibres of approximately 5,000/yr [13].

Blood flow to the anterior optic nerve is autoregulated by endovascular and metabolic factors similar to cerebral circulation. Autoregulation is effective over a critical range of perfusion pressure - not clearly defined in humans. Diseases of the circulatory system (hypertension, diabetes mellitus, atherosclerosis) damage autoregulation and therefore increase the critical perfusion pressure below which autoregulation of the anterior optic nerve fails. PION arises from the suboptimal blood supply from branches of the ophthalmic artery and the central retinal artery. The risk factors for both AION and PION in non-surgical patients include: high serum cholesterol and triglycerides, lipidaemia, fibrinogenaemia, DM and smoking. Risk factors in cardiac surgical patients include: intrinsic small vessel disease (DM), hyperglycaemia, hypertension, prolonged hypotension, vasospasm secondary to inotropes, the inflammatory process induced by CPB, anaemia and lower than normal cup:disc ratio which correlates with small scleral canal and possibly emboli [1]. A combination of risk factors in a patient may lead to additive or synergistic effects on blood flow and autoregulation.

A large retrospective time-matched, case-control study of 27,915 patients who underwent CPB was undertaken using the Mayo Clinic Surgical and Medical Indexes [6]. ION was identified in 17 (0.06%) cases. 2 controls for each ION patient were chosen by selecting 2 patients who underwent CPB exactly 2 weeks before each ION patient. They found significant risk factors for post-operative ION following cardiac surgery using CPB to be: advanced age, lower minimum post-operative haemoglobin concentrations, history of clinically severe vascular disease, pre-operative angiogram within 48 hours of CPB, longer pump times, surgical disruption of particulate matter, RBC transfusions and use of non-RBC blood components. Visual field and acuity loss was present in 100% of the cases and no improvement was found on follow-up.

Anaemia is an amenable risk factor to efficient correction. Nevertheless optimum haemoglobin concentration is a controversial subject especially in patients with cardiovascular disease. By contrast Spiess found that a higher haematocrit on intensive care admission was associated with Q wave MI [15]. Brown et al reported a series of 6 patients over a 10-year period that developed AION related to anaemia and hypotension after various surgical procedures [16]. There is debate as to whether cardiac patients behave similarly to other surgical patients [12]. Roth investigated the effect of isovolaemic haemodilution on ocular blood flow in cats [17]. He found a 71% increase in retinal blood flow and retinal O_2 _delivery remained approximately constant as the increased blood flow countered a significant decrease in arterial O_2 _content. Neely et al found feline retinal tissue oxygen tension increased initially during isovolaemic haemodilution to 50% above baseline at about 2/3 of the original haematocrit level and beyond this a steady decline in tissue oxygen tension [18]. Among cardiac surgical patients Mansour carried out a retrospective chart review of 1594 consecutive CABG patients over a five-year period (1995-1999) in one medical centre [19]. 3 patients experienced visual loss due to anterior ischaemic optic neuropathy - all had diabetes mellitus and 2 suffered severe postoperative anaemia. Among diabetics the risk of anterior ischaemic optic neuropathy was significantly higher in subjects with a postoperative haematocrit below 22% compared with the non-diabetic population (28.6% v 0.21%, p = 0.001).

Increasing the transfusion threshold in an effort to correct anaemia may minimize the chances of ION. However, transfusion in cardiac surgery has been shown to increase morbidity and mortality. Engoren demonstrated that transfused cardiac surgical patients had a five-year mortality double that of non-transfusion group [20]. Similarly Kudavalli found increased one-year mortality in the transfusion group [21]. Habib found increased incidence of renal injury and Banbury found increased risk of infection after transfusion in cardiovascular surgery [22,23]. Surgenor found that treatment of anaemia with transfusion increased the risk of low output cardiac function requiring treatment with inotropes and intra-aortic balloon counter pulsation [24]. In Hebert's multicentre RCT, he found no significant difference in 30-day or 60-day mortality in ICU patients with significant cardiovascular disease whether restrictive or liberal transfusion triggers were used [14]. However restrictive transfusion triggers appeared to be associated with significantly lower mortality in younger patients with lower APACHE scores.

So, with regard to correction of anaemia it would seem that we are "damned if we do and damned if we don't - withholding transfusion may have a cost in terms of increased incidence of ION or renal failure but transfusion itself may increase morbidity and mortality [25]. The Society of Thoracic Surgeons and the Society of Cardiovascular Anaesthesiologists recommend transfusion thresholds >7 g.dl^-1 ^[26].

Given the concerns surrounding transfusion as a treatment for perioperative anaemia, it has been proposed that prophylactic measures to minimize bleeding might be the solution to the problem. In a meta-analysis by Laupacis, tranexamic acid administration was associated with lower transfusion requirements in patients undergoing cardiopulmonary bypass [27]. Nevertheless, a major concern with tranexamic acid use is the possibility of increased risk of thrombembolic events, however the evidence is weak at present [28].

Another risk factor for ION, which may be amenable to intervention, is patient positioning. Prone position is known to increase IOP and although prone positioning is not used routinely in cardiac surgical patients, they may develop a lung injury in the post-operative period either from the cardiopulmonary bypass itself, transfusion, or sepsis and I feel it is worth a mention. Ozcan looked at the effect of prone positioning in awake volunteers and found IOPs 50% higher in prone horizontal patients [29]. The rise in IOP could be reduced with head-up tilt of 10 degrees, but not to normal. Cheng found IOP doubled in prone anaesthetised patients [30]. The episcleral venous system is connected to the central venous system via a valveless arrangement such that an increase in CVP causes an increase in episcleral venous pressure and hence increased IOP. There have been many reports of ION following surgical procedures performed in the prone position. Proning is also increasingly used in intensive care to treat patients with ARDS and the effect on IOP should also be considered in these critically ill patients. A mild degree of reverse Trendelenberg may ameliorate the increased episcleral venous pressure caused by proning. There are as yet no published cases of blindness associated with proning in ARDS, but it is only a matter of time. We are proposing to carry out a survey of practice across hospitals in the geographical area in order to establish practice with regard to proning in ITU and actions taken to defend ocular perfusion. We also wish to carry out a preliminary crossover study measuring intraocular pressure and haemodynamic parameters in supine position before proning procedure and thereafter in prone flat and then with a moderate degree of head-up position.

Perfusion pressure of the anterior optic nerve is the difference between the ciliary artery and venous drainage - approximately MAP - IOP. Increased IOP may substantially reduce the perfusion pressure of the anterior optic nerve. Increased IOP may contribute to the higher incidence of ION after operations involving CPB. Sanjay et al showed that intraocular pressure (IOP) increases on commencement of CPB and maximally at 20 minutes to 21 mmHg [31]. Methods to reduce IOP and therefore maximise intraocular perfusion pressure must be sought, including head position and use of loose endotracheal tube ties. CVP can increase beyond IOP and affect intraocular perfusion as it is a valveless system. This is in addition to external pressure on the globe [12]. Visual loss is not always associated with intraoperative hypotension, however it is quoted in a number of case reports [5,12]. Often the nadir of perfusion pressure is no different between those affected and unaffected after CPB and a modicum of hypotension is a frequent occurrence in anaesthetised patients, which may be associated with altered autoregulation or anatomical variation of optic nerve blood supply [12,32,33].

Embolic phenomena are another possible cause of ION and may be related to aortic cross clamping [34]. In a prospective study of neurological complications of coronary artery bypass surgery in 312 patients, 25.6% developed neuro-ophthalmological complications [35]. Horton showed visible retinal emboli in three patients, two coronary artery bypass graft patients using CPB and one cardiac catheterisation patient [36]. Intra-operative epi-aortic ultrasound may be a solution to minimise embolic risk for placement of the aortic cross-clamp. A "no-touch" technique may be used in off-pump CABG and alternative route CPB [37]. A sutureless proximal aortic anastamotic technique has been shown to reduce micro-embolic counts on transcranial Doppler [38].

The use of inotropes has also been implicated in cases of ION. Shapira et al found the development of AION in 8 patients out of 602 consecutive cardiac surgery patients (1.3%), an increase from their usual 0.5% rate [33]. Although it was impossible to implicate any single factor in the increased incidence, prolonged CPB, lower haematocrit, and peri-operative use of adrenaline and amrinone were all found to be associated. In one case series of 4 patients with AION occurring within a one-month period, high doses of mixtures of inotropes were one of the putative causes [39]. Adrenaline is postulated to cause severe vasospasm of the ocular vessels [40,41]. This presents anaesthetists with a difficult clinical decision since prolonged hypotension is also listed as a risk factor for ION.

In conclusion perioperative ION is a rare, devastating event, which may be difficult to detect in a sedated post-operative patient. The signs are subtle and are often late to present. Cardiac surgical procedures involving CPB appear to be at increased risk of causing this condition especially if complicated by hypotension, use of inotropes or anaemia. No effective treatment for ION exists, once established, hence prevention is crucial. Despite this, studies to date have only succeeded in demonstrating that minimizing one risk factor for the development of ION may merely serve to introduce another.

## Consent

Written informed consent was obtained from the patient for publication of this case report and any accompanying images. A copy of the written consent is available for review by the Editor-in Chief of this journal.

## Competing interests

The authors declare that they have no competing interests.

## Authors' contributions

BT, LMcW and AFP wrote the paper. BT and LMcW performed the literature search. HG reviewed all drafts of the manuscript and suggested changes. BV, SAP and RG added important comments to the paper. All authors have read and approved the final manuscript.
